# Impact of obesity on endometrial blood flow in women without polycystic ovarian syndrome during intracytoplasmic sperm injection

**DOI:** 10.1186/1477-7827-11-57

**Published:** 2013-06-26

**Authors:** Xun Zeng, Houqing Pang, Xiaohong Li, Shan Luo, Song Jin, Shangwei Li

**Affiliations:** 1 Reproductive Medical Center, West China Second University Hospital, Sichuan University, Chengdu, People’s Republic of China; 2 Ultrasound department, West China Second University Hospital, Sichuan University, Chengdu, People’s Republic of China

**Keywords:** Endometrial and subendometrial blood flow, Obesity, Power Doppler, Intracytoplasmic sperm injection

## Abstract

**Background:**

Obesity may exert a negative effect on in vitro fertilization (IVF)/intracytoplasmic sperm injection (ICSI) treatment. However, the effect of obesity on the endometrium remains unknown. This study was designed to assess the effect of isolated body mass index (BMI) on endometrial blood supply in non-polycystic ovary syndrome (PCOS) women during ICSI by power Doppler Ultrasound.

**Methods:**

An observational prospective study was carried out. A total of 206 patients without PCOS were divided into 4 groups based on Chinese BMI classification (kg/m(2): underweight (BMI < 18.5), normal weight (18.5 less than or equal to BMI < 24), overweight (24 less than or equal to BMI < 28), and obese (BMI greater than or equal to 28). Endometrial thickness, endometrial pattern, endometrial spiral arterial resistance index (RI) and pulsatility index (PI) values and systolic/diastolic ratio (S/D) were assessed on the day of human chorionic gonadotropin administration.

**Results:**

Obese patients required more doses of gonadotrophin and longer stimulation duration than the normal weight patients (P < 0.05). Endometrial thickness and pattern were not statistically different between the 4 BMI subgroups (P > 0.05). Subendometrial blood flow was detected in 165 (80.1%) patients and spiral arterial PI was significantly higher in the obese group than in the normal weight and underweight groups (P < 0.05). All parameters of ICSI outcome were comparable, including pregnancy and miscarriage rates.

**Conclusions:**

Obesity (BMI greater than or equal to 28 kg/m(2)) appears to exert a negative effect on endometrial and subendometrial blood flow based on the Chinese standard of obesity; however, it seems to have no significant effect on ICSI outcomes in non-PCOS women.

## Background

Obesity is a chronic condition characterized by an accumulation of body fat. In recent years, the reproductive ability of the obese population has become a growing concern. The adverse effect of obesity on natural fecundity including subfertility (increased time to conception) [1-3] and infertility [4,5], has been reportedly attributed to oligoovulation or anovulation.

The impact of obesity on in vitro fertilization (IVF) outcome is controversial. Some studies reported that obese women experience longer ovarian stimulation and require more gonadotropin ampoules [6] but respond poorly, with a decrease in the number of oocytes [6-8] and poorer outcomes, including impaired embryo quality, lower fertilization rate, and increased miscarriage rate [9,10]. However, no such effect has been noted in other studies [11,12].

The effect of obesity on the endometrium has received less attention. The endometrium is the vascular mucosal lining of the uterus. Apart from endometrial thickness and pattern, an adequate endometrial blood supply is an essential requirement for implantation [13]. Advances in power Doppler ultrasonography allowed in vivo assessments of the endometrium, including morphologic (thickness and pattern) and functional (vascularization) parameters. Power Doppler is particularly useful for minute vessels with low-velocity flow and is less dependent on the angle between the beam and vessel; in areas of multiple vessels, the signal is additive, and opposite flow directions do not cancel each other's signals [14].

The sonographic endometrial region is defined as the area between both hyperechogenic margins of the endometrium, whereas the sonographic subendometrial region is considered a thin hypoechogenic layer at the myometrial-endometrial junction between the myometrium and endometrium on ultrasound [15]. Related studies found that the subendometrial region plays an important role in endometrial function [16]. Sufficient blood supply to the endometrium and subendometrium indicates good endometrial receptivity, and an absence of both is representative of an unfavorable uterine environment. Furthermore, lower spiral artery pulsatility index (PI) at the beginning of treatment or on the oocyte pick-up (OPU) day was found in pregnancy cycles compared to nonpregnancy cycles, whereas the spiral artery resistance index (RI) and systolic/diastolic ratio (S/D) did not differ between the groups along the course of treatment [17,18].

A lack of evidence remains as to whether the body mass index (BMI) of infertile patients affects the endometrial blood flow during IVF. In Chinese women, the current World Health Organization’s (WHO) definition of adult overweight (BMI > 25 kg/m^2^) and obesity (BMI > 30 kg/m^2^) may not be applicable. It had been suggested that BMI cut-off point for overweight or obesity for the Chinese population should be lower than that for the North American or European populations because obesity-associated metabolism is lower in Chinese individuals than in North American or European populations [19,20]. Therefore, we adopted the Chinese BMI cut-off values proposed by the Working Group on Obesity in China (WGOC) [21] and described in the Guidelines for Prevention and Control of Overweight and Obesity in Chinese Adults [22] to define overweight or obesity, as follows: BMI < 18.5 kg/m^2^ (underweight), 18.5 ≤ BMI < 24 kg/m^2^ (normal weight), 24 ≤ BMI < 28 kg/m^2^ (overweight), and BMI ≥ 28 kg/m^2^ (obese). Because of the aforementioned concerns, we conducted this study in non-polycystic ovary syndrome (PCOS) women and assessed the effects of isolated BMI on the endometrial and subendometrial blood supply using the power Doppler technique during intracytoplasmic sperm injection (ICSI) cycles.

## Methods

Two hundred and thirty-one consecutive women undergoing ICSI at the Reproductive Center of West China Second Hospital, Sichuan University between March 2011 and January 2012 were enrolled in our prospective cohort study. Each patient provided written informed consent before participating in the study, which was approved by the Ethics Committee of West China Second University Hospital of Sichuan University.

The inclusion criteria were as follows: age, <35 years, basal serum follicle stimulating hormone (FSH <10 mIU/mL) level tested on day 2–3 of a spontaneous cycle within 2–3 months before IVF treatment, regular menstrual cycle (26–33 days), and no PCOS. To exclude the effect of previous abortions and any possible interference from previous stimulation, only the first cycle for each patient without previous pregnancies was analyzed. The exclusion criteria were as follows: evidence of endometrial anomalies, anovulation, hypothalamic amenorrhea, and current or past diseases such as hepatic, renal, adrenal, or thyroid disorders that affected the ovaries or gonadotropin or sex steroid secretion or excretion.

Twenty-five women were excluded from the study. Six (2.6%) had poor ovarian response, thus OPU was not performed. Fresh embryo transfer (ET) was cancelled in 14 women including 5 (2.2%) with moderate to severe ovarian hyperstimulation, no embryo transferred was encountered in 4 (1.7%) women, 3 (1.3%) with a progesterone level of > 3 pg/uL on the day of human chorionic gonadotropin (hCG) administration, and 2 (0.9%) with fluid accumulation in the uterine cavity observed on ultrasound on ET day. Five (2.2%) women withdrew their informed consent. Finally, 206 consecutive patients were evaluated prospectively.

The height and weight of each patient was obtained to calculate the BMI immediately prior to treatment. Patients were categorized according to BMI as follows: underweight group (BMI < 18.5 kg/m^2^), normal weight group (18.5 ≤ BMI < 24 kg/m^2^), overweight group (24 ≤ BMI < 28 kg/m^2^), and obese group (BMI ≥ 28 kg/m^2^).

All patients received a standard long protocol, starting with pituitary downregulation with a gonadotropin-releasing hormone agonist (GnRH-a, Decapeptyl, Ferring AG) during the mid-luteal phase. Then, ovarian stimulation was performed using recombinant follicle-stimulating hormone (rFSH, Gonal F, Serono), starting from days 2–3 of the next cycle. The initial daily FSH dose was adjusted for age, ovarian volume, and antral follicle count, and varied between 150 and 300 IU. Subsequent gonadotropin doses were adjusted based on the ovarian response as assessed by estradiol levels and follicular growth. 10,000 IU of urinary hCG was administered intramuscularly for ovulation induction when 2 or more follicles were >18 mm in diameter. Oocyte retrieval was performed 35–37 h later by ultrasonographic-guided transvaginal puncture. Standard procedures were performed for gamete-embryo handling. Day 3 ET was performed in all cases. The embryos were graded on day 3 according to the following 3 characteristics: the form of the blastomeres, percentage of fragmentation, and cleavage rate. Luteal support was then given. Clinical pregnancy was defined as a sonographic visualization of intrauterine gestational sac 5 weeks after ET.

All patients underwent a transvaginal sonography (TVS) by a single ultrasound practice doctor who specialized in gynecological imaging. The ultrasound machine was an Acuson Sequoia 512 ultrasound system (Siemens, Mountain View, CA, USA). All ultrasound images were digitally stored and re-reviewed by a single reviewer (Author Song Jin) who specialized in gynecological sonography. Because of the reports of increased impedance of uterine arteries after hCG injection and its influence on the predictive value of ultrasonography [23], ultrasonography was performed on the day of hCG injection around 8–10 am after the patients had emptied their bladder. Endometrial thickness was measured from the echogenic interface of the junction of the myometrium and endometrium through the central longitudinal axis of the uterus. The endometrial pattern was classified as either a triple layer or non-triple layer. A triple layer endometrium presented a pattern in which the hyperechogenic outer lines and central hyperechogenic line were observed with a hypoechogenic region between them. A non-triple layer consisted of homogenous endometrial patterns characterized by either hyperechogenic or isoechogenic endometrium.

Using power Doppler with 6.0 MHz ultrasonography in the two-dimensional (2-D) mode in selected areas, endometrial blood flow was detected by endometrial or adjacent subendometrial regions within 10 mm of the echogenic endometrial borders. The endometrial-subendometrial blood flow distribution pattern was determined by the presence of pulsatile color signals in the subendometrial and endometrial regions. The endometrial-subendometrial blood flow distribution pattern [24] was classified as follows: Group A : No detectable endometrial or subendometrial flow; Group B: Presence of subendometrial flow only; and Group C: Presence of endometrial and subendometrial flow. If subendometrial flow was observed, Doppler studies were performed on the spiral artery with the highest color intensity within the innermost endometrial-subendometrial area in the upper part of the proximal uterine wall, obtained in the sagittal uterine plane. After confirming that the waveforms were continuous, an average of 3–5 cardiac cycles was selected for calculation of RI, PI, and S/D. Uterine circulation was assessed simultaneously in each examination; bilateral uterine arteries were sampled lateral to the cervix near the internal orifice. Mean values of bilateral uterine RI and PI were used for analysis.

### Statistical analysis

Continuous variables were presented as the mean ± standard deviation (SD) if normally distributed or given as the median (interquartile range) when not normally distributed. Analysis of variance (ANOVA) was applied to compare normally distributed data using the Bonferroni test for multiple post hoc analyses. Variables that were not normally distributed were analyzed using the Kruskal-Wallis test and the Mann–Whitney *U* test where appropriate. Chi-square and Fisher’s exact tests were used to compare categorical data as appropriate. The Statistical Package for the Social Sciences 13.0 software (SPSS Inc., Chicago, IL, USA) was used for data analysis. P < 0.05 (two tailed) was considered statistically significant.

## Results

Two hundred and six patients undergoing fresh autologous IVF/ICSI cycles were recruited: 42 in the underweight group, 80 in the normal weight group, 44 in the overweight group, and 40 in the obese group. All 4 BMI subgroups were comparable with regard to baseline characteristics including age, infertility duration, and day 3 FSH level (Table 1).

**Table 1:** **Baseline characteristics and ovarian response in non-PCOS women (n = 206) according to BMIs (kg/m**^**2**^**) in ICSI**

	**BMI < 18.5**	**18.5 ≤ BMI < 24**	**24 ≤ BMI < 28**	**BMI ≥ 28**	**P**
*No*. *of patients*	42	80	44	40	-
*Age (y)*	28.9 ± 4.5	29.2 ± 4.1	29.0 ± 3.9	30.5 ± 4.0	0.830
*Infertility duration (y)*	5.1 ± 2.5	4.9 ± 2.7	5.8 ± 3.1	5.5 ± 3.0	0.532
*Day 3 FSH (mIU/mL)*	5.8 ± 2.2	5.5 ± 1.8	5.9 ± 2.0	6.2 ± 1.9	0.426-
*Recombinant FSH dosage (IU)*	2013.5 ± 771.6	1996.4 ± 884.2	2433.2 ± 997.4	2952.7 ± 1158. 2	<0.05 ^c,d,e,f^
*Length of stimulation (days)*	8.2 ± 2.7	8.1 ± 2.4	9.9 ± 2.2	11.5 ± 3.9	<0.05 ^c,d,e,f^
*Serum E2 (pg/ml)*^*b*^	2367.7 ± 998.2	2572.3 ± 892.5	2238.3 ± 866.9	2293.3 ± 905.1	0.340
*Mean No. of oocytes obtained*	10.0 ± 6.1	9.8 ± 5.7	9.5 ± 5.1	8.9 ± 5.9	0.291
*Mean No. of embryos transferred*	1.9 ± 1.0	2.0 ± 1.0	2.0 ± 0.9	1.9 ± 0.9	0.768
*Mean grade of transferred embryos*	1.9 ± 0.1	2.0 ± 0.2	2.1 ± 0.1	2.0 ± 0.1	0.523
*Pregnancy rate*	16/42 (38.1%)	36/80 (45.0%)	18/44 (40.9%)	14/40 (35.0%)	0.737
*Miscarriage rate*	2/16 (12.5%)	4/36 (11.1%)	3/18 (16.6%)	2/14 (14.2%)	0.951

Note: Values are mean ± SD.

^a^ Number of patients (%).

^b^ On the day of hCG.

^c^ P value obtained by group-to-group comparison, BMI < 18.5 kg/m^2^ vs. 24 ≤ BMI < 28 kg/m^2^.

^d^ P value obtained by group-to-group comparison, BMI < 18.5 kg/m^2^ vs. ≥28 kg/m^2^.

^e^ P value obtained by group-to-group comparison, 18.5 ≤ BMI < 24 kg/m^2^ vs. 24 ≤ BMI < 28 kg/m^2^.

^f^ P value obtained by group-to-group comparison, 18.5 ≤ BMI < 24 kg/m^2^ vs. ≥28 kg/m^2^.

The overweight and obese groups required higher doses of gonadotropin and longer stimulation duration compared to the underweight and normal weight groups (P < 0.05). The mean serum estradiol concentration on the day of hCG injection, mean number of oocytes obtained, and mean number of embryos transferred were comparable between the 4 BMI subgroups (P > 0.05).

The endometrial thickness, endometrial pattern, and uterine PI, RI, and S/D on the day of hCG injection were not statistically different between the 4 BMI groups (P > 0.05), although there was a trend toward decreased endometrial thickness as the BMI increased. For the distribution pattern of the endometrial-subendometrial blood flow on the day of hCG injection, the absence of endometrial and subendometrial flow (pattern A) was detected more frequently in the obese group than in the other 3 subgroups, although this difference did not reach statistical significance (P > 0.05) (Table 2).

**Table 2:** **Endometrial thickness, pattern and endometrial-subendometrial flow distribution pattern in women without PCOS (n = 206) according to BMIs (kg/m**^**2**^**)**

	**BMI < 18.5**	**18.5 ≤ BMI < 24**	**24 ≤ BMI < 28**	**BMI ≥ 28**	**P**
	**(n = 42)**	**(n = 80)**	**(n = 44)**	**(n = 40)**	
***Endometrial thickness (mm)***	10.6 ± 2.1	10.5 ± 2.3	10.1 ± 2.2	9.5 ± 2.9	0.055
***Endometrial pattern ***^***a***^					0.872
*Triple layer*	34 (81.0)	65 (81.3)	35 (79.5)	30 (75.0)	
*Non-triple layer*	8 (19.0)	15 (18.7)	9 (20.5)	10 (25.0)	
***Endometrial blood flow distribution pattern ***^***a***^					0.149
*A :subendometrial flow (−), endometrial flow (−)*	9 (21.4)	11 (13.8)	10 (22.7)	11 (27.5)	
*B :subendometrial flow (+), endometrial flow (−)*	13 (31.0)	22 (27.5)	13 (29.5)	17 (42.5)	
*C :subendometrial flow (+), endometrial flow (+)*	20 (47.6)	47 (58.7)	21 (47.7)	12 (30.0)	
***Uterine PI ***^***b***^	2.10	2.11	2.11	2.13	0.413
	(1.49-2.76)	(1.41-2.86)	(1.38-2.87)	(1.33-3.08)	
***Uterine RI ***^***b***^	0.80	0.81	0.82	0.82	0.683
	(0.63-0.87)	(0.72-0.89)	(0.71-0.87)	(0.73-0.92)	
***Uterine S/D ***^***b***^	5.69	5.71	5.70	5.89	0.561
	(4.33-6.89)	(4.50-6.92)	(4.61-7.03)	(4.23-6.95)	

Note: Values are mean ± SD.

*RI* resistance index, *PI* pulsatility Index, *S/D*  the ratio between peak systolic flow and lowest diastolic flow.

^a^ Number of patients (%).

^b^ Values given as median (range).

Subendometrial blood flow was detected in 165 (80.1%) patients on the day of hCG injection. In these patients, spiral arterial PI was significantly higher in the obese group compared to the normal weight and underweight groups (P < 0.05). However, the mean measurements of RI and S/D of the spiral arteries did not differ between the 4 groups (P > 0.05) (Table 3). No significant differences were observed in the pregnancy and miscarriage rates of the 4 BMI subgroups (P > 0.05) (Table 1).

**Table 3:** **Spiral flow parameters in women without PCOS whose subendometrial flow can be detected (n = 165) according to BMIs (kg/m**^**2**^**)**

	**BMI < 18.5**	**18.5 ≤ BMI < 24**	**24 ≤ BMI < 28**	**BMI ≥ 28**	**P**
	**(n = 33)**	**(n = 69)**	**(n = 34)**	**(n = 29)**	
*Spiral PI*	1.00 (0.80-1.39)	0.96 (0.75-1.45)	1.14 (0.78-1.55)	1.29 (0.77-1.62)	<0.05 ^a, b^
*Spiral RI*	0.59 (0.52-0.64)	0.60 (0.52-0.67)	0.61 (0.56-0.62)	0.61 (0.56-0.70)	0.483
*Spiral S/D*	2.91 (2.72-3.21)	2.90 (2.60-3.24)	2.95 (2.70-3.41)	3.02 (2.71-3.35)	0.382

Note: Values are median (range). *RI*  resistance index, *PI*  pulsatility Index, *S/D*  the ratio between peak systolic flow and lowest diastolic flow.

^a^ P value obtained by group-to-group comparison, BMI < 18.5 kg/m^2^ vs. ≥28 kg/m^2^.

^b^ P value obtained by group-to-group comparison, 18.5 ≤ BMI < 24 kg/m^2^ vs. ≥28 kg/m^2^.

## Discussion

To the best of our knowledge, this is the first study to use power Doppler ultrasound to evaluate the effect of BMI on endometrial and subendometrial blood flow in non-PCOS women during ICSI treatment using the Chinese standard of obesity. In the current study, a trend toward increased gonadotropin dose and stimulation duration was noted in obese women compared to normal weight women. In the patient whose subendometrial blood flow was detected, the RI and S/D measurements were comparable between the 4 BMI subgroups, although spiral arterial PI was significantly higher in the obese group than in the normal weight group. However, this did not translate to inferior pregnancy outcomes, as the clinical pregnancy and miscarriage rates were comparable between the 4 BMI groups.

Increasing evidence on the effect of obesity on IVF treatment has shown that increased BMI has adverse effects on ovarian stimulation response [8,10,25-27]. Li et al. [25] reported that overweight women (BMI ≥ 24 kg/m^2^) required more ampoules of gonadotropin, had lower peak E2 concentration along with increased cycle cancellation because of insufficient follicle development. Orvieto et al. [26] and Dokras et al. [27] reported similar results in obese women (BMI > 30 kg/m^2^). In the present study, we demonstrated that obesity increased the required gonadotropin dose and stimulation duration. The reason for gonadotropin resistance in obesity is unclear; however, alterations in serum and follicular concentrations of adipokine leptin might be involved [28]. Higher leptin levels can directly attenuate insulin, insulin-like growth factor-1, and transforming growth factor-β [29], which may adversely affect granulosa cell function [30] and thecal cell steroidogenesis, and simultaneously suppresses follicular growth by reducing sensitivity to gonadotropin stimulation [31]. The doses were not similarly initiated and maintained in the 4 BMI groups. Therefore, in women with lower ovarian response, gonadotropin doses and stimulation duration were increased; these women were frequently obese. Thus, despite the state of gonadotropin resistance in the obese group, similar serum estradiol concentrations on the day of hCG injection and comparable numbers of retrieved oocytes were achieved in 4 BMI subgroups.

Uterine receptivity is more important than is currently believed. Because of insufficient knowledge on the role of the endometrium in the reproductive performance of obese patients, our study focused primarily on the endometrial factor. Previous studies focusing on the effects of BMI on the endometrium only evaluated the association between endometrial thickness and BMI, demonstrating conflicting results. Ku et al. reported no significant difference in endometrial thickness on the day of hCG injection between women with BMI < 24 kg/m^2^ and those with BMI ≥ 24 kg/m^2^; however, the clinical pregnancy rate was lower in the latter [32]. Esinler et al. also reported comparable endometrial thickness in different BMI subgroups [33]. However, Nichols et al. noted that the mean endometrial thickness on the day of retrieval was increased in obese women (BMI ≥ 28 kg/m^2^), with a significant reduction in pregnancy rate compared to underweight (BMI < 20 kg/m^2^) or normal-weight women (BMI 20–27.9 kg/m^2^) [34]. In our study, the endometrial thickness and pattern on the day of hCG administration was not significantly different between the 4 BMI subgroups; however, a nonsignificant trend toward increased endometrial thickness was noted with higher BMI. Differences in the BMI cutoff values and inclusion criteria may be one of the reasons for these conflicting results. In our study, we use the Chinese BMI cut-off values and only the first cycle for each patient without previous pregnancies was analyzed; therefore, the influence of previous abortions and any possible interference from previous stimulation on the endometrial growth was avoided. However, the 3 aforementioned studies did not consider this factor.

A high degree of endometrial perfusion is an essential requirement for early endometrial response to blastocyst implantation, and vascular changes may affect uterine receptivity [35]. The significant increase in endometrial microvascular blood flow periovulation affects endometrial growth, blood supply, and implantation [13]. The presence of both endometrial and subendometrial blood flow on color or power Doppler examination has been used as an indicator of sufficient endometrial receptivity, whereas the absence of both represents a poor uterine environment. Zaidi et al. [36] assessed the power Doppler image of the endometrial strip on the day of hCG administration and showed that patients who did not develop both endometrial and subendometrial vascularization were associated with implantation failure. Similar conclusions were obtained by Chein et al. [24]. They reported that subendometrial blood flow was detected in 477 (76.6%) patients; pregnancy and implantation rates were significantly higher in these patients than in those with no detectable flow (P < 0.0001).

Increased impedance in the subendometrial arteries could be expected in cases of diminished endometrial vascularization and perfusion. Higher spiral arterial PI appears to negatively affect endometrial blood vessel function. Gorokhovsky et al. [17] assessed 39 women undergoing IVF and found significantly reduced spiral artery PI values in pregnancy cycles compared to nonpregnancy cycles at the beginning of treatment. Spiral artery RI and S/D did not differ between these groups along the treatment course. Similar conclusions for endometrial Doppler assessment of spiral artery RI and S/D were reached by Battaglia et al. and they demonstrated a significantly lower PI in spiral arteries on the day of OPU in pregnancy cycles compared with nonpregnancy cycles [18]. However, another study found no difference in the measurements of PI, RI, and S/D between pregnant and nonpregnant patients [14].

No data are available concerning the effects of BMI on endometrial perfusion in women during IVF cycles. In the present study, we measured resistance to flow in the endometrium using the Power Doppler technique and noted more frequent absence of endometrial and subendometrial flow on the day of hCG injection in the obese group than in the other 3 subgroups, although this difference did not reach statistical significance. Subendometrial blood flow was detected in 165 (80.1%) patients on the day of hCG injection. In these patients, the spiral arterial PI was significantly higher in the obese group than in the normal weight group, but the RI and S/D measurements were comparable in the 4 BMI subgroups. The significant difference in PI, but not in RI and S/D, could be related to the PI calculation method, which takes the entire spectral Doppler waveform into account and not just the maximal and minimal frequency shifts, as in the calculation of RI and S/D [17]. Sivitz et al. [37] reported that microvascular vasodilator function is impaired in obese women, and women appeared more sensitive to the deleterious effect of obesity on endothelium-mediated resistance vessel function. Obesity appears to negatively affect endometrial blood supply because of the higher PI value in obese women than in the controls.

Despite the lack of knowledge on the role of the endometrium in the reproductive performance of obese patients, most reports show lower implantation and pregnancy rates and higher miscarriage rates [5,9,10]. However, other authors have not found a detrimental effect of increased BMI on IVF/ICSI outcomes [12,33]. Dechaud et al. [12] examined 573 patients who underwent 789 IVF cycles and found that all parameters of IVF outcome were comparable between different BMI subgroups, including the cancellation, implantation, and pregnancy rates, with the exception of significantly increased required r-FSH dose in the obese group. A retrospective study of 775 patients who underwent 1113 ICSI cycles showed that endometrial thickness was comparable in different BMI subgroups, and isolated high BMI (≥30 kg/m^2^) only negatively affected ovarian stimulation response and availability of cryopreservable embryos [33]. In our study, the pregnancy and miscarriage rates did not differ between the 4 BMI subgroups. This finding could be explained mainly by the lack of differences in the number of embryos transferred and the mean grade of transferred embryos in our study, as the poor embryo quality described in obese women has been the main parameter related to increased risk of implantation failure and miscarriage [38,39].

Several caveats should be considered when comparing the results of the effect of obesity on IVF outcome. The lack of consensus regarding the impact of obesity on ovarian response, endometrial thickness and pattern, clinical pregnancy rate, and miscarriage rate might be partly explained by the different definitions of normal weight, overweight, or obesity as defined by the BMI values used to create study groups. The distribution of BMI differs according to ethnicity and environment. It has been demonstrated that Chinese individuals have similar distributions of glucose and lipid factors at significantly lower BMI values compared with European individuals [19]. The WHO criteria derived in European populations may not apply to Chinese populations. We adopted different BMI cutoff limits largely derived from Chinese population studies demonstrating that a BMI of 18.5–23.9 kg/m^2^ should be considered “normal,” a BMI between 24.0 and 27.9 kg/m^2^ considered “overweight,” and a BMI of ≥28.0 kg/m^2^ considered “obese” [22]. Therefore, the validity of the current results is dependent on the Chinese classification of BMI used in our study.

Our study was a prospective, observational trial with a homogenous sample in which the ovarian stimulation protocol and some confounding factors such as age, the first ICSI cycle, and PCOS were taken into consideration. There is a current worldwide epidemic of both obesity and PCOS, and PCOS is frequently associated with increased BMI. Furthermore, PCOS is also the most common cause of anovulatory infertility and is associated with many adverse ART outcomes. However, the majority of studies with conflicting results in this context do not discriminate between isolated obesity and PCOS. Therefore, it is essential to exclude such patients in order to delineate the effect of isolated obesity on ICSI outcome, as in the present study.

## Conclusions

According to the Chinese classification of BMI, obese women required more doses of gonadotrophin and longer stimulation duration than normal weight women. Isolated obesity had a negative impact on endometrial and subendometrial blood flow. However, this did not translate to inferior pregnancy outcomes, since the clinical pregnancy and miscarriage rates were comparable between all BMI subgroups. Further studies should explore other morphologic, sonographic, and endocrine parameters to determine the associations between obesity, the endometrium, and IVF outcomes.

## Competing interests

The authors declare that they have no competing interests.

## Authors’ contributions

XZ conceived and drafted the manuscript. HQP designed the study and performed ultrasound examination. XHL designed the study and performed statistical analysis of data. SL participated in the data collection and drafted the manuscript. SJ participated in the ultrasound image collection and interpreted the data. ZW supervised the team and the study. All authors read and approved the final manuscript.
